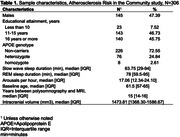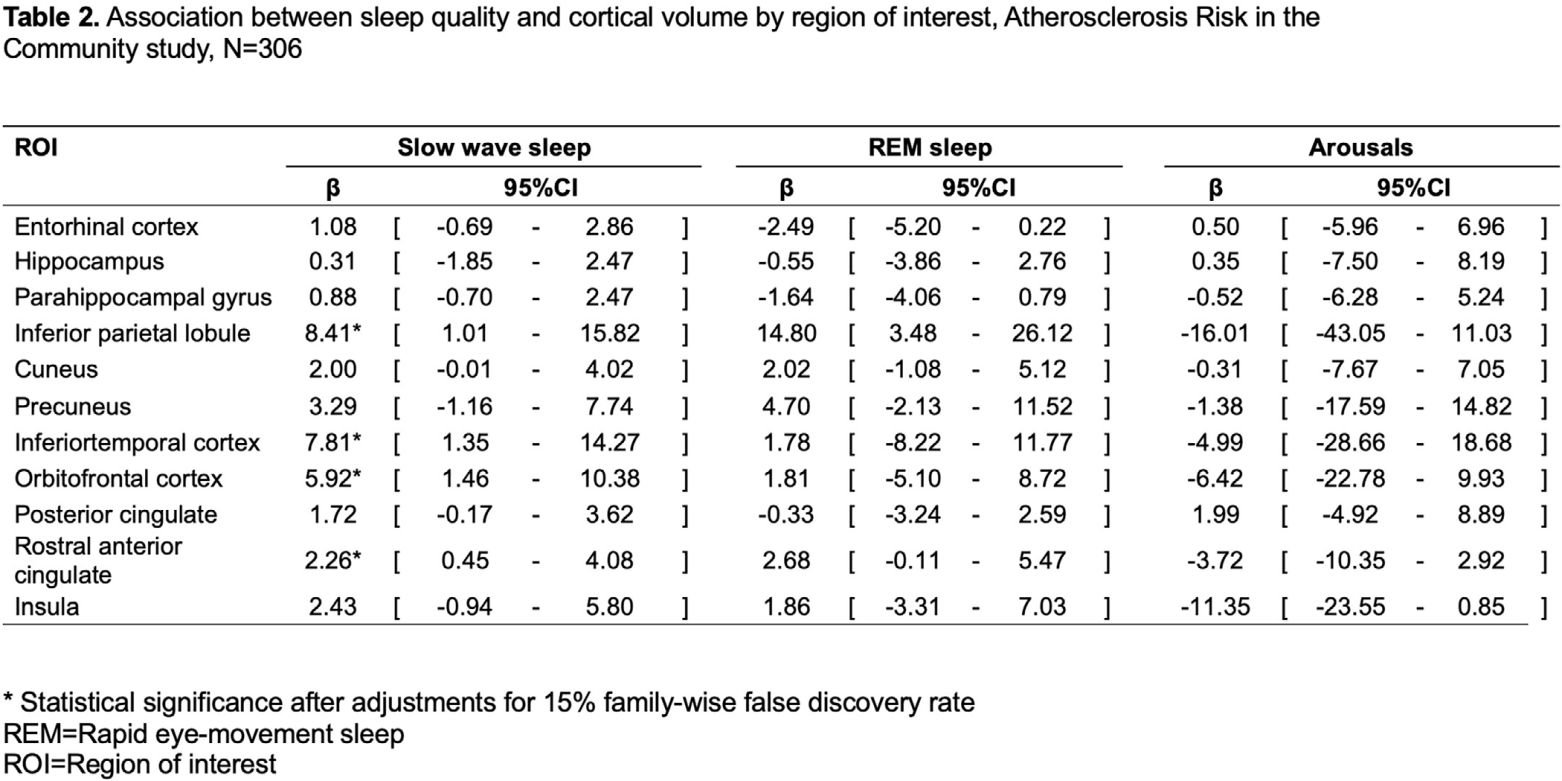# Lower slow wave sleep and rapid eye‐movement sleep are associated with brain atrophy of AD‐vulnerable regions

**DOI:** 10.1002/alz.093827

**Published:** 2025-01-09

**Authors:** Gawon Cho, Adam P Mecca, Orfeu M. Buxton, Xiao Liu, Brienne Miner

**Affiliations:** ^1^ Yale School of Medicine, New Haven, CT USA; ^2^ Alzheimer’s Disease Research Unit, Yale University School of Medicine, Department of Psychiatry, New Haven, CT USA; ^3^ Pennsylvania State University, University Park, PA USA

## Abstract

**Background:**

Sleep deficiency is associated with an increased risk of Alzheimer’s disease (AD), warranting research on underlying mechanisms. This study examined the association of sleep architecture with anatomical features frequently observed in AD: (1) atrophy of cuneus, hippocampus, entorhinal, inferior parietal, parahippocampal, and precuneus regions (henceforth referred to as “AD‐vulnerable regions”) and (2) the presence of cerebral microbleeds.

**Method:**

In 271 participants of the Atherosclerosis Risk in the Communities Study, we examined the prospective association of baseline sleep architecture with anatomical features of the brain identified on MRI conducted ∼17 years later. Sleep architecture was quantified as the proportion of time spent in slow wave sleep (SWS), proportion of time spent in rapid eye‐movement sleep (REM), and the number of arousals per hour of total sleep time using polysomnography. Outcomes included (1) volumetric measurements of each AD‐vulnerable region and (2) the presence of any cerebral microbleeds (CMBs) and that of lobar CMBs, which are more specifically associated with AD. We analyzed the association of each sleep predictor with each brain outcome, adjusting for demographics, baseline cognitive function, *APOE* genotype, intracranial volume, other covariates, and false discovery rate (FDR; family‐wise *p*<.05).

**Result:**

Having less SWS was associated with smaller volumes of the inferior parietal region (β = ‐41.56 mm^3^ per percentage point (PP) [95%CI = ‐73.97, ‐9.15]) and cuneus (β = ‐11.56 mm^3^ per PP [‐20.51,‐2.62]) after adjustments for covariates. Having less REM was associated with smaller volumes of the inferior parietal region (β = ‐73.13 mm^3^ per PP [‐126.95, ‐19.30]) and precuneus (β = ‐32.46 mm^3^ per PP [‐64.48,‐0.44]). After FDR adjustments, lower SWS and REM, respectively, were associated with lower volumes of the inferior parietal region only. Arousal index was not associated with the volumes of AD‐vulnerable regions. None of the sleep architecture variables were associated with the presence of CMBs or lobar CMBs.

**Conclusion:**

Sleep deficiency is associated with the atrophy of the inferior parietal region, which is often observed in early AD. Sleep architecture may be a modifiable risk factor for AD pathogenesis.